# Attenuated Risk Association of End-Stage Kidney Disease with Metformin in Type 2 Diabetes with eGFR Categories 1–4

**DOI:** 10.3390/ph15091140

**Published:** 2022-09-13

**Authors:** Aimin Yang, Eric S. H. Lau, Hongjiang Wu, Ronald C. W. Ma, Alice P. S. Kong, Wing Yee So, Andrea O. Y. Luk, Amy W. C. Fu, Juliana C. N. Chan, Elaine Chow

**Affiliations:** 1 Department of Medicine and Therapeutics, The Chinese University of Hong Kong, Prince of Wales Hospital, Hong Kong SAR 999077, China; 2 Hong Kong Institute of Diabetes and Obesity, The Chinese University of Hong Kong, Prince of Wales Hospital, Hong Kong SAR 999077, China; 3 Asia Diabetes Foundation, Hong Kong SAR 999077, China; 4 Li Ka Shing Institute of Health Sciences, The Chinese University of Hong Kong, Prince of Wales Hospital, Hong Kong SAR 999077, China; 5 Hong Kong Hospital Authority Head Office, Hong Kong SAR 999077, China; 6 Phase 1 Clinical Trial Centre, The Chinese University of Hong Kong, Prince of Wales Hospital, Hong Kong SAR 999077, China

**Keywords:** metformin, diabetes, cardiovascular disease, end-stage kidney disease, lactic acidosis, mortality

## Abstract

Type 2 diabetes (T2D)-associated end-stage kidney disease (ESKD) is a global burden, while the renoprotective effects of metformin remain controversial. In a population-based cohort (2002–2018) including 96,643 patients with T2D observed for 0.7 million person-years, we estimated the risk association of metformin and its dose-relationship with ESKD in a propensity-score overlap-weighting (PS-OW) cohort by eGFR categories. Amongst 96,643, 83,881 (86.8%) had eGFR-G1/G2 (≥60 mL/min/1.73 m^2^), 8762 (9.1%) had eGFR-G3a (≥45–60 mL/min/1.73 m^2^), 3051 (3.2%) had eGFR-G3b (≥30–45 mL/min/1.73 m^2^), and 949 (1.0%) had eGFR-G4 (≥15–30 mL/min/1.73 m^2^). The respective proportions of metformin users in these eGFR categories were 95.1%, 81.9%, 53.8%, and 20.8%. In the PS-OW cohort with 88,771 new-metformin and 7872 other oral glucose-lowering-drugs (OGLDs) users, the respective incidence rates of ESKD were 2.8 versus 22.4/1000 person-years. Metformin use associated with reduced risk of ESKD (hazard ratio (HR) = 0.43 [95% CI: 0.35–0.52] in eGFR-G1/G2, 0.64 [0.52–0.79] in eGFR-G3a, 0.67 [0.56–0.80] in eGFR-G3b, and 0.63 [0.48–0.83] in eGFR-G4). Metformin use was associated with reduced or neutral risk of major adverse cardiovascular events (MACE) (7.2 versus 16.0/1000 person-years) and all-cause mortality (14.6 versus 65.1/1000 person-years). Time-weighted mean daily metformin dose was 1000 mg in eGFR-G1/G2, 850 mg in eGFR-G3a, 650 mg in eGFR-G3b, and 500 mg in eGFR-G4. In a subcohort of 14,766 patients observed for 0.1 million person-years, the respective incidence rates of lactic acidosis and HR in metformin users and non-users were 42.5 versus 226.4 events/100,000 person-years (*p* = 0.03) for eGFR-G1/G2 (HR = 0.57, 0.25–1.30) and 54.5 versus 300.6 events/100,000 person-years (*p* = 0.01) for eGFR-G3/G4 (HR = 0.49, 0.19–1.30). These real-world data underscore the major benefits and low risk of lactic acidosis with metformin use down to an eGFR of 30 mL/min/1.73 m^2^ and possibly even 15 mL/min/1.73 m^2^, while reinforcing the importance of dose adjustment and frequent monitoring of eGFR.

## 1. Introduction

Metformin is the first-line oral glucose-lowering drug (OGLD) in type 2 diabetes (T2D) used for over 60 years [1]. A lack of randomized clinical trial (RCT) data and reports of lactic acidosis have raised concerns regarding its use in patients with reduced kidney function [2]. With increasing real-world evidence (RWE) supporting its safety in patients with broad range of kidney function, in 2016, the United States Federation Drug Administration requested the manufacturer to change the label, allowing its use in patients with estimated-glomerular filtration rate (eGFR) 30–60 mL/min/1.73 m^2^ (G3) [3].

Metformin possesses glucose-lowering, insulin-sparing, anti-inflammatory and anti-fibrotic properties, with possible organ-protective effects [4,5,6,7]. In 2018, a meta-analysis of 40 studies including 1 million patients reported 20–40% reduced risk for cardiovascular (CV), all-cause mortality, and CV-events with metformin use, although data on reno-protection remained inconsistent [8]. In a retrospective cohort of 10,426 patients with T2D [9], metformin users had 35% reduced risk for all-cause mortality and ESKD, especially in those with eGFR ≥ 30–45 mL/min/1.73 m^2^. However, lack of adjustment for time-varying metformin exposure and HbA1c and eGFR, which were both confounders and mediators, introduced major biases [10]. Despite the large sample size, most meta-analyses included patients with T2D and eGFR ≥ 30 mL/min/1.73 m^2^ followed up for less than 5 years. Biases due to incomplete reporting of baseline and time-varying covariates or dosage [10,11], as well as insufficient adjustment for discontinuation of metformin and immortal bias due to intervening events [9,12,13,14], are other limitations.

The Kidney Disease Improving Global Outcomes (KDIGO) practice guidelines classified kidney function by eGFR (G1: ≥90, G2: 60–89, G3a: 45–59, G3b: 30–44, G4: 15–29, and G5: <15 mL/min/1.73 m^2^). Metformin, mainly renally excreted, inhibits the mitochondrial respiratory chain (MRC1) with a theoretical risk of increased lactic acidosis, especially in the setting of hypoxia with increased anaerobic metabolism, increased production with sepsis, and/or reduced renal clearance [15]. In 2020, experts recommended continuation of metformin in patients with eGFR-G3 by halving the maximum dose and increasing the frequency of eGFR monitoring in patients with eGFR-G3b [16].

Diabetes-associated end-stage kidney disease (ESKD) [17] is a huge healthcare burden, which is calling for urgent evidence regarding the safety and effectiveness of metformin as a low-cost medication for possible renoprotection. RWE using informative databases analysed by appropriate methodologies addressing multiple biases provide important insights [10,18,19]. In this real-world study, we evaluated the benefits and risk of metformin use in patients with reduced (eGFR-G3/G4) versus preserved (eGFR-G1/G2) kidney function [18] and risk of lactic acidosis using a territory-wide population-based cohort and a register-based cohort.

## 2. Results

### 2.1. Baseline Characteristics

In the territory-wide HKDSD cohort including 753,374 patients with diabetes in 2019, 20.4% patients had eGFR-G3 at enrolment, 3.3% had eGFR-G4, and 1.8% had eGFR-G5. Since not all patients in the HKDSD had undergone structured assessment, we used the comprehensive data in the RAMP module to curate a cohort using predefined inclusion/exclusion criteria to avoid bias of prevalent use and disease severity consisting of 96,643 patients followed up for a mean (SD) of 6.9 (4.0) years (665,267 person-years). At baseline, 83,881 (86.8%) had eGFR-G1/G2, 8762 (9.1%) had eGFR-G3a, 3051 (3.2%) had eGFR-G3b, and 949 (1.0%) had eGFR-G4. The respective proportions of metformin users were 95.1%, 81.9%, 53.8%, and 20.8%. During the follow-up, there were 88,771 (91.9%) new-metformin and 7872 (8.1%) other-OGLDs users. New-metformin users had a shorter diabetes duration and were less likely to be treated with sulfonylurea and RASis than other-OGLDs users. Following PS-OW, all characteristics were well-balanced between both groups (Table 1).

### 2.2. Metformin Use and Risk of ESKD

**Figure 1:**
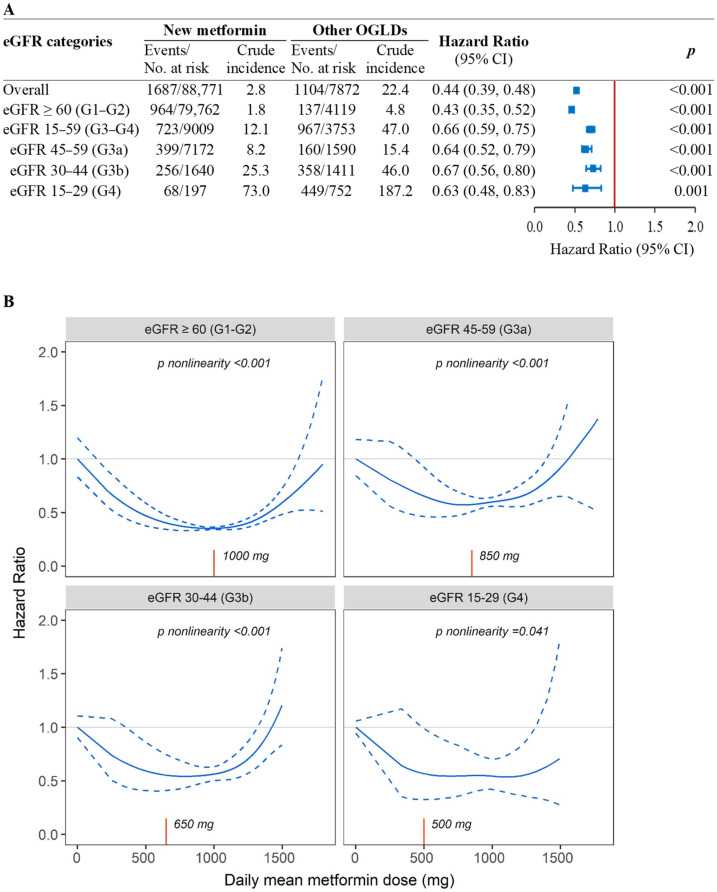
Associations of new-metformin use versus other-OGLDs use (**A**) and mean daily dose of metformin (**B**) with risk of ESKD by eGFR categories in the population-based cohort. (**A**) Results were yielded using fixed-time Cox model with adjustment for baseline covariates due to confounding effect of eGFR on metformin use in the new-user population-based cohort with propensity-score overlapping weight. (**B**) Penalized spline curve analyses with 3 knots were performed (*n* = 96,643) using time-fixed Cox model with daily mean dose of metformin exposure. Both analyses were adjusted for confounding effects due to age, sex, disease duration, HbA1c, lipids, cardiometabolic risk factors, cancer history, and use of diabetes-related medications including insulin, oral glucose-lowering drugs (OGLDs) (sulfonylureas, thiazolidinediones, dipeptidyl-peptidase-4 inhibitors [DPP-4is], alpha-glucosidase inhibitors [AGIs], glucagon-like peptide-1 receptor analogue [GLP-1RAs], sodium-glucose co-transporter 2 inhibitors [SGLT2is]), RAS inhibitors, and statins at enrolment and during follow-up. Time-weighted mean daily metformin dose (red *X*-axis line) was 1000 mg in G1-G2 (*n* = 79,762), 850 mg in G3a (*n* = 7172), 650 mg in G3b (*n* = 1640), and 500 mg in G4 (*n* = 197).

In the PS-OW matched cohort, the crude incidence rates of ESKD in the new-metformin users versus other-OGLDs users was 2.8 versus 22.4 events/1000 person-years, with metformin use associated with reduced risk of ESKD across all eGFR categories (HR [95% CI]: 0.43 [0.35–0.52] in eGFR-G1/G2, 0.64 [0.52–0.79] in eGFR-G3a, 0.67 [0.56–0.80] in eGFR-G3b, and 0.63 [0.48–0.83] in eGFR-G4) (Figure 1A). In the spline analysis, the time-weighted mean daily metformin dose was non-linearly associated with ESKD in eGFR-G1/G2 (*P_nonlinearity_* < 0.001), eGFR-G3a (*P*_nonlinearity_ < 0.001), eGFR-G3b (*P_nonlinearity_* < 0.001), and eGFR-G4 (*P_nonlinearity_* = 0.041). The time-weighted mean daily metformin dose was estimated to be 1000 mg in eGFR-G1/G2 (*n* = 79,762), 850 mg in eGFR-G3a (*n* = 7172), 650 mg in eGFR-G3b (*n* = 1640), and 500 mg in eGFR-G4 (*n* = 197) (Figure 1B).

### 2.3. Metformin Use and Lactic Acidosis

**Figure 2:**
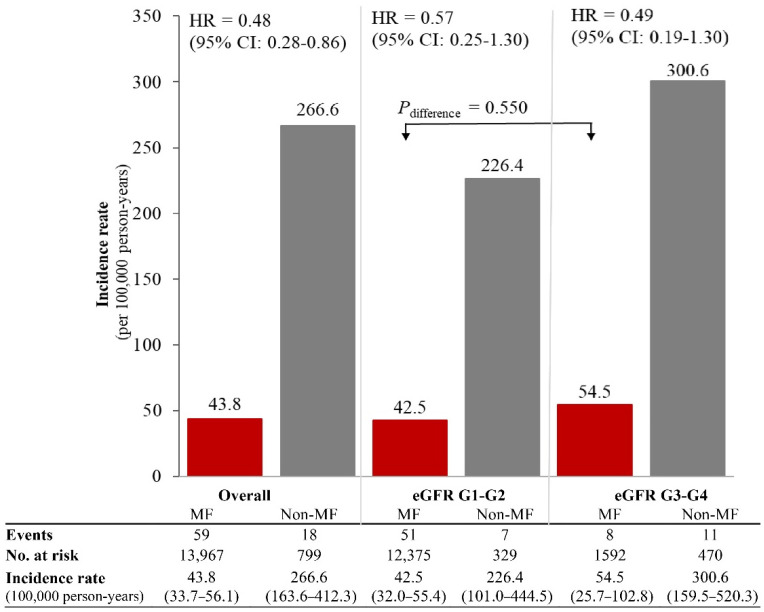
Rates of lactic acidosis amongst metformin (MF) users and non-metformin users by eGFR categories in the register-based cohort. The rates of lactic acidosis amongst metformin users (*n* = 13,967) and non-metformin users (*n* = 799) were calculated in the register-based cohort (*n* = 14,766), stratified by eGFR categories. Hazard ratios (HR) and 95% confidence interval (CI) of lactic acidosis associated with metformin use versus non-metformin use were estimated using time-fixed Cox model adjusted for age, sex, disease duration at enrollment, time-varying exposure to sulfonylureas and insulin, and index year of enrolment.

In the HKDR, 14,766 patients (13,967 metformin and 799 non-metformin users) were followed up for a mean (SD) of 9.6 (4.1) years (141,310 person-years). Metformin users had shorter duration of diabetes, higher BMI and eGFR, and lower urine ACR than non-metformin users (Table S2). There were 77 lactic acidosis events (54.5 [95% CI: 43.3–67.7] events/100,000 person-years) including 59 events in 13,967 metformin-users and 18 events in 799 non-metformin users. Metformin users had a lower incidence of lactic acidosis than non-metformin users for eGFR-G1/G2 (42.5 [32.0–55.4] versus 226.4 [101.0–444.5] events/100,000 person-years, *P_difference_* = 0.03) and eGFR-G3/G4 (54.5 [25.7–102.8] versus 300.6 [159.5–520.3] events/100,000 person-years, *P_difference_* = 0.01) (Figure 2). Metformin use was associated with reduced risk of lactic acidosis with HR of 0.48 (0.27–0.86) overall. On subgroup analysis, similar risk associations were observed in eGFR-G1/G2 (HR = 0.57, 0.25–1.30) and in eGFR-G3/G4 (HR = 0.49, 0.19–1.30). Amongst metformin users, there was no difference in the incidence of lactic acidosis between eGFR-G1/G2 and eGFR-G3/G4 (*P_difference_* = 0.550).

### 2.4. Metformin Use and Risk of All-Cause Mortality and MACE

**Figure 3:**
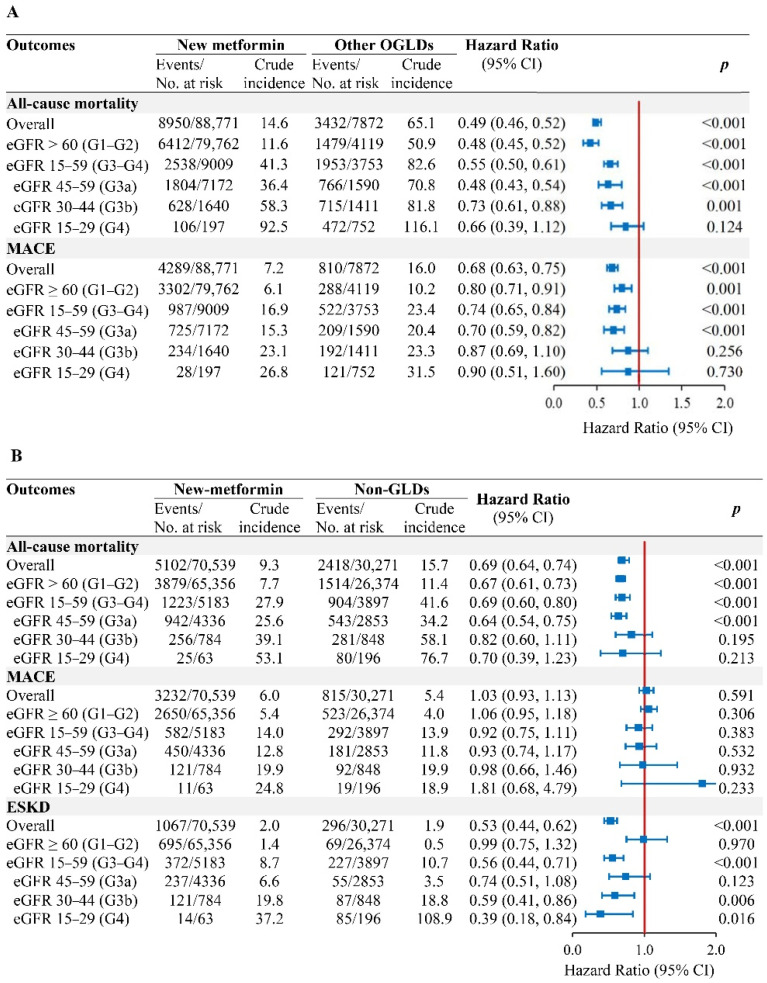
Associations of new-metformin use versus other-OGLDs use (**A**) and non-GLDs use (**B**), with risk of outcomes by eGFR categories in the population-based cohort. In the new-user population-based cohorts with propensity-score overlapping weight, Cox model of all-cause mortality and MACE with time-varying metformin exposure, adjusted for covariates including HbA1c, lipids, comorbidities (CVD and cancer), use of diabetes-related medications including insulin, OGLDs (sulfonylureas, thiazolidinediones, dipeptidyl-peptidase-4 inhibitors [DPP-4is], alpha-glucosidase inhibitors [AGIs], glucagon-like peptide-1 receptor analogue [GLP-1RAs], sodium-glucose co-transporter 2 inhibitors [SGLT2is]), statins, and RAS inhibitors during follow-up. Results of ESKD were yielded using fixed-time Cox model with adjustment for baseline covariates due to confounding effect of eGFR on metformin use.

In the population-based PS-OW cohort, the respective crude incidence rates of all-cause mortality and MACE in new-metformin versus other-OGLDs users were 14.6 versus 65.1 and 7.2 versus 16.0 events/1000 person-years (Figure 3A). Metformin use was associated with reduced or neutral risk for all-cause mortality (HR [95% CI]: 0.48 [0.45–0.52] in eGFR-G1/G2, 0.48 [0.43–0.54] in eGFR-G3a, 0.73 [0.61–0.88] in eGFR-G3b, and 0.66 [0.39–1.12] in eGFR-G4). The respective HR of MACE were 0.80 (0.71–0.91), 0.70 (0.59–0.82), 0.87 (0.69–1.10), and 0.90 (0.51–1.60) (Figure 3A). The metformin dose relationship was non-linear with all-cause mortality in eGFR-G1/G2, eGFR-G3a, and eGFR-G3b (all *P_nonlinearity_* < 0.001) but not in eGFR-G4 (*P_linearity_* = 0.160) (Figure S3). For MACE, non-linearity was observed for eGFR-G1/G2 (*P*_nonlinearity_ < 0.001) and eGFR-G4 (*P_nonlinearity_* = 0.046) but not in eGFR-G3a (*P*_nonlinearity_ = 0.071) and eGFR-G3b (*P*_linearity_ = 0.600).

### 2.5. Sensitivity Analysis

In the new-metformin (*n* = 70,539) versus non-GLDs (*n* = 30,271) PS-OW cohort, 91,730 (91.0%) had eGFR-G1/G2, 7189 (7.1%) had eGFR-G3a, 1632 (1.6%) had eGFR-G3b, and 259 (0.3%) had eGFR-G4 (Table S3). The respective incidence rates of ESKD, all-cause mortality, and MACE in new-metformin versus non-GLDs users were 2.0 versus 1.9, 9.2 versus 15.7, and 6.0 versus 5.4 events/1000 person-years (Figure 3B). Metformin use was associated with reduced risk of ESKD in eGFR-G3b/G4 (HR = 0.56, 0.44–0.71), all-cause mortality in eGFR-G1/G2 (HR = 0.67, 0.61–0.73), and eGFR-G3a (HR = 0.64, 0.54–0.75), with neutral risk of MACE across all eGFR categories.

## 3. Discussion

In this comprehensive analysis, we asked an important question, whether metformin, as a low-cost medication, could prevent ESKD in patients with T2D in different eGFR categories. Diabetes-associated ESKD has a major impact on healthcare costs and quality of life [17]. There is a large body of RWE supportive of the neutral or beneficial effects of metformin on MACE and all-cause mortality, but its protective effects on kidney function remain inconclusive. To our knowledge, this is the largest prospective cohort analysis showing that 50% of patients with eGFR-G3 and 20% of patients with eGFR-G4 were treated with metformin. To avoid confounding due to indication bias and disease severity, we only included new-metformin users and compared their outcomes with patients treated with other OGLDs and non-GLDs. Using different models, we confirmed that metformin use was associated with 33–57% lower risk of ESKD in all eGFR categories (G1-G4) versus other OGLDs. In the comparison between metformin and non-GLDs users, the rate of ESKD was low, although the reduced risk association with metformin remained significant in those with eGFR-G3b/G4. In a register-based analysis and based on review of medical records, lactic acidosis was a rare event with metformin use being associated with lower risk versus non-metformin use. Amongst metformin users, the rate of lactic acidosis was similar in eGFR-G1/G2 and eGFR-G3/G4.

In Asia, 10–40% of patients receiving out-patient diabetes care had chronic kidney disease (CKD) with eGFR-G3 or less [19]. Consistent with the popularity of metformin, half of the patients (53.8%) were treated with metformin in eGFR-G3 and 20.8% in eGFR-G4 in the current study. In Germany and Australia, 15% of patients with T2D and eGFR-G3 were prescribed with metformin [20,21]. Given the fact that metformin is renally excreted and less frequently prescribed in patients with CKD, few studies had included sufficiently large number of patients with reduced kidney function to address the risk–benefit ratio of metformin use in these high-risk patients [14]. While there is consistent RWE on reduced risk of mortality in patients treated with metformin [22], there are conflicting reports on its associations with MACE and ESKD, especially in patients with eGFR-G4/G5.

In a post hoc analysis of the TREAT (Trial to Reduce Cardiovascular Events With Aranesp Therapy) Trial, which compared the use of erythropoietin versus a placebo in 4038 patients, with T2D-associated CKD and anaemia, followed up for 29.1 months, metformin was associated with lower risk of ESKD in eGFR-G1/G3 (HR = 0.70, 0.53–0.92), but neutral risk in eGFR-G4/G5 (HR = 0.95, 0.70–1.29) [12]. In another retrospective cohort of 10,426 patients, followed up for 7.3 years, the risk of metformin with ESKD was attenuated or insignificant in 208 patients with eGFR <45 mL/min/1.73m^2^ or lower [9]. Subsequently, these results had been criticized for lack of adjustment for prevalent bias and metformin discontinuation [10]. Our results closed this knowledge gap using data from 3051 patients with eGFR-G3b and 949 patients with eGFR-G4. These renoprotective effects in all eGFR categories down to 15 mL/min/1.73 m^2^ aligned with the modulating effects of metformin on inflammation, oxidative stress, and dysregulation of microbiota, which are implicated in CKD [23]. Metformin inhibits mitochondrial metabolism with a reduced ADP/ATP ratio, which activates AMP kinase with reduced endogenous glucose production. Of note, only 80% of metformin are absorbed, and the interaction between metformin and gut microbiota in modulating the inflammatory and redox milieu is now considered an important mechanisms for its multi-system effects [1].

In the register-based analysis (*n* = 14,766), we performed detailed review of medical records to ascertain the occurrence of lactic acidosis. The low incidence of 42.5 (32.0–55.4) and 54.5 (25.7–102.8) events/100,000 person-years in patients with eGFR-G1/G2 and eGFR-G3/G4 were similar to another report with 41.8 (36.3–42.7) events/100,000 person-years in patients with CKD [24]. In our analysis, within the same eGFR category, there was no difference in lactic acidosis rates between metformin and non-metformin users. Amongst the metformin users, the rate of lactic acidosis was also similar between patients with preserved or reduced kidney function. In another retrospective study lasting for 5.7 years, time-varying metformin use was not associated with increased risk of lactic acidosis in eGFR-G3 (*n* = 9093), albeit with increased risk in patients with eGFR-G4/G5 (*n* = 1579) (HR = 2.07, 1.33–3.22) [25]. In our study, there were 949 patients with eGFR-G4, and we did not include patients with eGFR-G5.

Dose adjustment of metformin is recommended with declining kidney function. In our spline analysis, prolonged exposure to metformin was associated with reduced or neutral risk of ESKD in all eGFR categories down to eGFR-G4. The time-weighted mean daily dose was estimated to be 850 mg (eGFR-G3a), 650 mg (eGFR-G3b), and 500 mg (eGFR-G4). These dosages were lower than the recommended doses of 1500 mg (eGFR-G3a) and 1000 mg (eGFR-G3b) by KDIGO [16]. Our results were more akin to that of 1500 mg, 1000 mg, and 500 mg in eGFR-G3a, G3b, and G4, with no accumulation of lactic acid as reported in a pharmacokinetic study [26]. In a study involving 813 metformin and 2439 non-metformin users, followed up for 2.1 years, metformin use was associated with increased risk of mortality in patients with ESKD (HR = 1.4, 1.2–1.5) [13]. In our study, we did not include patients with eGFR-G5, which was the outcome measure.

Our study had both strengths and limitations. Real-world evidence generated from high quality databases analysed by appropriate methodology can complement RCT data to identify unmet needs, adverse events, and unanticipated benefits of interventions including medications [27]. These RWE data are particularly important in the absence of RCT data for generating hypothesis, designing experiments, and informing practice guidelines [28]. Our study had the largest number of patients with eGFR-G3b (*n* = 3015) and eGFR-G4 (*n* = 949), with a mean follow-up period of 6.9 years. The detailed documentation of baseline and time-varying covariates allowed implementation of robust methodology including new-user design and multiple modelling to adjust for different biases. This contrasts previous metformin-based analyses, often biased due to incomplete or random data retrieved from administrative databases [10,18,19]. Using baseline data collected during structured assessment and time-varying data from a territory-wide EMR, we used PS-OW matching to create a cohort mimicking that of RCT. We excluded patients treated with insulin or prior events, which only represented ~15% of the original cohort, making our results generalizable to the majority of patients. We excluded patients already exposed to metformin for removing indication basis and confirmed new-metformin users had reduced risk of ESKD, in patients down to eGFR-G4 compared to non-users. Out study also had limitations, which included non-randomized nature, unmeasured covariates (e.g., prescribers’ preference and patients’ adherence), and residual confounding inherent with all observational studies. Plasma metformin levels were not measured in routine practice. In agreement with other reports [29], the majority of lactic acidosis events were concluded as being unrelated to metformin.

## 4. Materials and Methods

### 4.1. Setting and Patients

Hong Kong has a population of 7.5 million, mainly of Chinese descent, with universal health coverage through care provision by the government-funded Hospital Authority (HA). The HA operates all hospitals and clinics with on-site drug dispensing, which have shared a territory-wide electronic medical records (EMR) system since 2000. The research group based at the Prince of Wales Hospital (PWH), the teaching hospital of the Chinese University of Hong Kong (CUHK), first introduced a research-driven quality improvement program in 1995, where patients were referred from medical clinics to undergo protocol-driven assessment by trained nurses at the Diabetes Centre, including eye, feet, blood, and urine examination, to identify care gaps. With patients’ consent, these data formed the basis of the Hong Kong Diabetes Register (HKDR) for research purposes [30]. In 2000, this protocol was adopted by the HA in a territory-wide Risk Assessment and Management Program for Diabetes Mellitus (RAMP-DM) in primary- and secondary-care settings [30].

In 2020, we curated data from the HA-EMR system to form the territory-wide Hong Kong Diabetes Surveillance Database (HKDSD) for research purposes [31]. Within the HKDSD, we extracted data from the RAMP-DM module, which captured data collected during the structured assessment [31]. We also reviewed medical records from the PWH-EMR for patients enrolled in the HKDR, to determine the occurrence of lactic acidosis (not available in the HKDSD). This study was approved by the Joint NTEC-CUHK Clinical Research Ethics Committee. This study is reported according to the Strengthening the Reporting of Observational Studies in Epidemiology (STROBE) guideline for cohort study.

### 4.2. Population-Based Cohort (HKDSD RAMP-DM Module)

**Figure 4:**
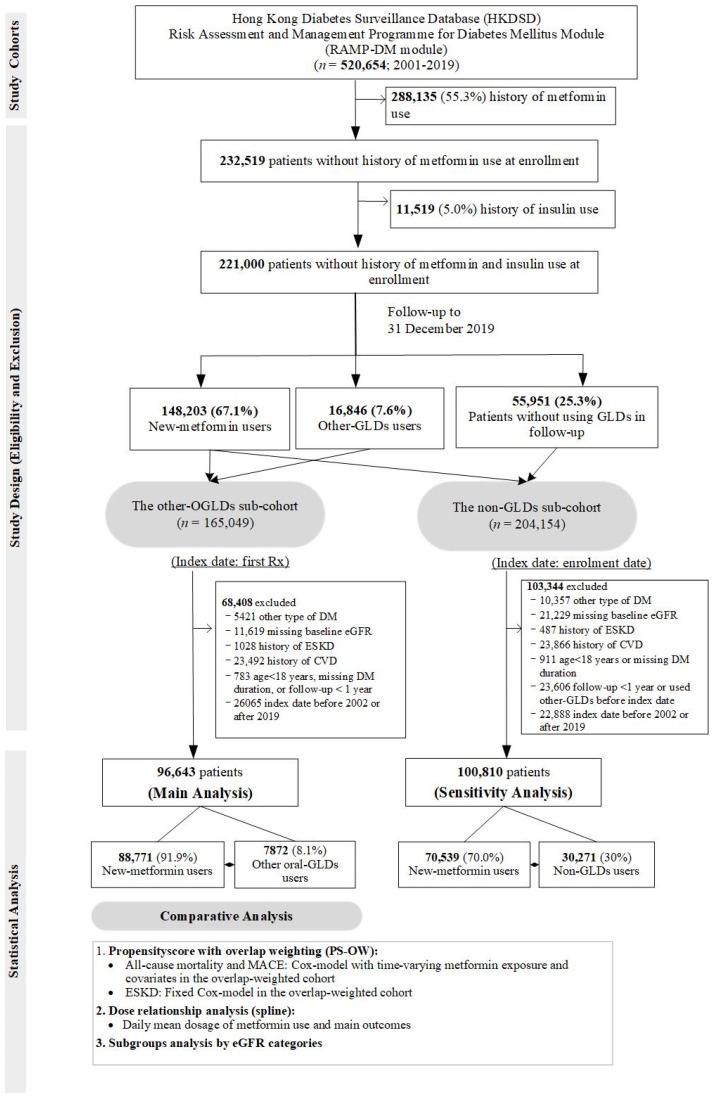
Study design and patient selection in the population-based cohort.

From the HKDSD RAMP-DM module (2001–2019), we curated a prospective cohort of 520,654 patients with T2D, defined as non-ketotic presentation and non-requirement of continuous insulin treatment within 12 months of diagnosis [32]. We excluded patients with exposure to metformin (*n* = 288,135) and insulin (*n* = 11,519) at enrollment to reduce prevalent bias and bias due to disease severity [28,33]. To overcome indication bias, we adopted a new-user design [34] in the remaining 221,000 patients subdivided into (1) new-metformin users versus other-OGLDs users in the main analysis and (2) new-metformin users versus patients not using any GLDs during the observation period (non-GLDs users) in the sensitivity analysis (Figure 4). We referred the index date to the first date of dispensing of metformin or other OGLDs (Figure S1A). We used 1-year period before index date as baseline and excluded patients with prior CVD and ESKD to address time-lag bias due to disease severity [28]. The follow-up period started at index date and ended at the earliest date of ESKD and events of interest or censor date, giving 96,643 patients (88,771 new-metformin and 7872 other-OGLDs users) for analysis (Figure 4).

### 4.3. Register-Based Cohort (PWH-Based HKDR Cohort)

In the HKDR, we curated data from 20,941 adult patients aged ≥18 years with T2D enrolled in 2000–2016, observed until 31 December 2019. The metformin-group included 13,967 patients, with 10,049 treated with metformin at enrolment and 3918 new-metformin users after enrolment. We excluded patients with ESKD (*n* = 311) or prior CVD (*n* = 4375), observed for less than one year (*n* = 206), or never exposed to any GLDs (*n* = 1283), giving 13,967 metformin users and 799 non-metformin GLDs users for analysis (Figure S2).

### 4.4. Ooutcomes Defintions

We used the principal discharge diagnosis in International Classification of Diseases, 9th Revision (ICD-9) and death codes (ICD-10) as well as laboratory values to define baseline and time-varying covariates in the RAMP-DM module and the HKDR. We used ICD-9 and ICD-10 codes to define the primary outcome of ESKD. This included dialysis or kidney replacement therapy (ICD-9 code) (Table S1) or eGFR < 15 mL/min/1.73 m^2^ on at least two occasions separated by ≥90 days. We excluded eGFR values measured during hospitalization with acute kidney injury based on ICD-9 code [16]. Other outcomes included all-cause mortality, major adverse cardiovascular events (MACE,) and its components including nonfatal acute myocardial infarction (AMI), ischemic heart disease (IHD), nonfatal stroke, and CV-death as well as hospitalizations due to heart failure based on ICD-9 codes (Table S1). All laboratory data including HbA1c, plasma glucose, lipids, and eGFR as calculated by the CKD-EPI Equation [35] were extracted from the EMR system.

In the HKDR cohort, we reviewed all medical records with events fulling the definition of lactic acidosis based on laboratory values (serum lactate > 5.0 mmol/L with a concomitant blood pH < 7.35) during the observation period [36]. Lactic acidosis events separated by more than one month were regarded as separate events.

### 4.5. Metformin Exposure and Other Medications Assessment

The HKDSD included dispensing data of diabetes-related medications including name, dose, frequency, duration (days), and start and end dates from 2000 to 2019. All medications were coded according to the Anatomical Therapeutic Chemical (ATC) code [32,37] including metformin, insulin, other OGLDs (sulfonylureas, thiazolidinediones, dipeptidyl-peptidase-4 inhibitors [DPP-4is], alpha-glucosidase inhibitors [AGIs], glucagon-like peptide-1 receptor analogue [GLP-1RAs], and sodium-glucose co-transporter 2 inhibitors [SGLT2is]), statins, and renin-angiotensin-system inhibitors (RASi) (Table S1). Fixed-dose combination formulations were counted as two different medications based on the active ingredient. Time-varying exposure to metformin and other medications were based on start and end dates of dispensing records within each follow-up year for each patient. We calculated the proportion of metformin exposure time based on the proportion of dispensing period in each year and the mean proportion of time exposure expressed against the total follow-up time. We calculated the time-weighted mean daily dose of metformin for each patient based on the mean daily dose dispensed during the follow-up period.

### 4.6. Time-Fixed and Time-Varying Covariates

Baseline covariates included clinical and laboratory data collected during the structured assessment in the RAMP-DM module and HKDR including socio-demographic profile, history of cancer, clinical (blood pressure, body mass index [BMI], waist circumference [WC]) and laboratory values: HbA1c, lipids (triglyceride [TG], low-density lipoprotein cholesterol [LDL-C], high-density lipoprotein cholesterol [HDL-C], total cholesterol [TC]), urine albumin-creatinine-ratio (ACR) and eGFR [30]. From the territory-wide EMR, we retrieved all laboratory (glucose, lipids and eGFR), comorbidities (hospitalization due to renal, CVD, and cancer events defined by ICD-9 and ICD-10 codes), and dispensing records as time-varying covariates during the baseline and observation period (Table S1).

### 4.7. Statistical Analysis

All data are expressed as mean (standard deviation, SD), median (interquartile range, IQR), and count (percentages). Student’s *t*-test, chi-squared, or analysis of variance (ANOVA) were used for group comparisons.

In the population-based cohort, we performed risk analysis on ESKD, MACE, and all-cause mortality in new-metformin versus other-OGLDs users after propensity-score overlap weighting (PS-OW) matching to homogenize baseline data [38]. We calculated the PS using a multivariate logistic regression model, and used the effect size of covariates selected based on prior knowledge and observations during clinical practice, to assign weights to balance all attributes at baseline for each patient using the OW approach [39] (Table 1). Compared with the classic PS methods of matching and inverse probability of treatment weighting, OW had better performance with respect to target population, balance, and precision [38].

In the PS-OW matched cohort, we performed Cox model with time-varying metformin exposure to adjust for discontinuation/switching of metformin and other time-varying covariates including HbA1c, lipids, use of other OGLDs, insulin, statins, RASi, and occurrence of CVD and cancer during follow-up [10]. For ESKD analysis, we used fixed-time Cox model due to the confounding effect of eGFR on metformin use. We conducted subgroup analyses with separate PS-OW matching stratification by baseline eGFR categories: ≥60 (G1/G2) and 15–59 (G3/G4) with subgroup analysis in patients with eGFR 45–59 (G3a), 30–44 (G3b), and 15–29 (G4) mL/min/1.73 m^2^, expressed as hazard ratio (HR) with 95% confidence interval (CI).

We calculated time-weighted mean daily metformin dose by eGFR categories, and incidence rates of ESKD and outcomes of interest expressed as 100,000 person-years. We estimated the relationships of time-weighted mean daily metformin dose with outcomes by eGFR categories using penalized spline curve in Cox model adjusted for age, sex, disease duration, cardiometabolic risk factors, and cancer history at enrolment as well as time-varying covariates as previously described. For ESKD, due to confounding effect of kidney function on metformin use, time-fixed Cox model was used. Likelihood-ratio test was used to select the spline models with 3 knots.

In the register-based cohort, we calculated incidence rates of lactic acidosis in metformin and non-metformin users by baseline eGFR categories (G1/G3 and G3/G4), expressed as 100,000 person-years, and estimated the HR (95% CI) of lactic acidosis associated with metformin in time-fixed Cox model adjusted for age, sex, disease duration, exposure to metformin, sulfonylureas, insulin, and index year.

### 4.8. Sensitivity Analysis

We excluded patients ever exposed to any GLDs, except for metformin and those with prior CVD and ESKD at enrollment, and repeated the analysis in 100,810 patients (70,539 new-metformin and 30,271 non-GLDs users) (Figure S1B). We applied Cox model and estimate risk association of metformin with ESKD and events of interests in the PS-OW matched cohort of new-metformin users versus non-GLDs users.

We checked for violation of assumption of proportional hazards using scaled Schoenfeld residual plots [40]. We handled missing data (missing rate < 15%) for time-varying covariates (HbA1c, eGFR and lipids) using multiple imputations by age, sex, and duration of diabetes [41]. All analyzes were implemented using R software (Version 4.0.0, R Core Team, R Foundation for Statistical Computing, Vienna, Austria). We used *PSweight* and *survey* packages to fit the PS-OW model and Cox models. A two-sided *p* value of <0.05 was considered statistically significant.

## 5. Conclusions

In real-world practice, metformin use was associated with reduced risk of ESKD and lactic acidosis, in patients with T2D with advanced CKD. These RWE underscores the major benefits and safety of metformin use down to an eGFR of 30 mL/min/1.73 m^2^ and possibly even 15 mL/min/1.73 m^2^, while reinforcing the importance of dose adjustment and frequent monitoring of eGFR. Large-scale RCTs have confirmed the renoprotective effects of SGLT2is in patients, with advanced CKD with or without T2D down to eGFR 30 mL/min/1.73 m^2^, albeit the majority of patients with T2D were treated with metformin during the trial period [42,43]. Many patients with T2D with preserved kidney function are now treated with metformin according to practice guidelines. Given the low cost of metformin, RCT comparing metformin versus other GLDs in patients with T2D and eGFR-G4 as well as patients with non-diabetes CKD will provide the definitive evidence regarding the renoprotective effect of metformin, which will have huge global impacts.

## Figures and Tables

**Table 1:** Characteristics of 96,643 patients in the population-based cohort with propensity-score overlap weighting (PS-OW).

Characteristics	New-Metformin Versus Other-OGLDs Users					
	Before PS-OW			After PS-OW		
	New-Metformin	Other-OGLDs	SMD	New-Metformin	Other-OGLDs	SMD
*n* (%)	88,771	7872		88,771	7872	
Men, %	41,531 (46.8)	4505 (57.2)	0.210	55.3	55.3	<0.001
Age, years	62.2 (11.4)	69.7 (11.8)	0.647	68.4 (12.1)	68.4 (11.0)	<0.001
Duration of diabetes, years	4.8 (5.3)	5.6 (6.1)	0.151	5.7 (6.3)	5.7 (5.2)	<0.001
Family history of diabetes	31,815 (35.8)	2107 (26.8)	0.197	28.4	28.4	<0.001
Smoking status			0.225			<0.001
Non-smoker	69,416 (78.2)	5544 (70.4)		72.6	72.6	
Ever-smoker	10,435 (11.8)	1566 (19.9)		17.7	17.7	
Current-smoker	8920 (10.0)	762 (9.7)		9.6	9.6	
Body mass index, kg/m^2^	26.1 (4.2)	25.1 (3.9)	0.237	25.2 (4.0)	25.2 (3.7)	<0.001
SBP, mmHg	133.5 (13.0)	137.7 (16.8)	0.280	136.4 (16.2)	136.4 (13.9)	<0.001
DBP, mmHg	74.8 (8.3)	72.4 (9.6)	0.258	72.8 (9.4)	72.8 (8.3)	<0.001
Waist, cm	89.5 (10.2)	88.7 (10.2)	0.082	88.6 (10.3)	88.6 (9.7)	<0.001
Triglyceride, mmol/L	1.8 (1.4)	1.6 (1.3)	0.102	1.6 (1.4)	1.6 (1.2)	<0.001
Total Cholesterol, mmol/L	5.0 (1.0)	4.9 (1.1)	0.101	4.9 (1.1)	4.9 (1.0)	<0.001
HDL-C, mmol/L	1.3 (0.4)	1.3 (0.4)	0.028	1.3 (0.4)	1.3 (0.4)	<0.001
LDL-C, mmol/L	2.9 (0.9)	2.9 (0.9)	0.062	2.9 (0.9)	2.9 (0.9)	<0.001
Urinary ACR, mg/mmol			0.367			<0.001
<3	64,808 (73.0)	4651 (59.1)		63.7	63.7	
3–30	20,368 (22.9)	2214 (28.1)		27.2	27.2	
>30	3595 (4.0)	1007 (12.8)		9.1	9.1	
HbA1c, %	8.0 (1.6)	7.1 (1.5)	0.585	7.3 (1.6)	7.3 (1.0)	<0.001
eGFR, mL/min/1.73 m^2^	84.1 (17.6)	62.6 (24.3)	1.017	68.6 (23.6)	68.6 (18.5)	<0.001
Haemoglobin, gm/dL	13.8 (1.5)	13.1 (1.9)	0.405	13.4 (1.8)	13.4 (1.7)	<0.001
History of cancer, %	3674 (4.1)	730 (9.3)	0.206	8.7	8.7	<0.001
History of medications, %						
Sulfonylurea	26,508 (29.9)	5663 (71.9)	0.928	64.1	64.1	<0.001
DPP-4is	149 (0.2)	77 (1.0)	0.108	0.6	0.6	<0.001
TZDs	59 (0.1)	30 (0.4)	0.067	0.3	0.3	<0.001
AGIs	121 (0.1)	48 (0.6)	0.078	0.5	0.5	<0.001
Statin	25,146 (28.3)	1527 (19.4)	0.211	20.9	20.9	<0.001
RASi	25,203 (28.4)	2950 (37.5)	0.194	35.2	35.2	<0.001
Period of index year, %			0.545			<0.001
<2003	2274 (2.6)	571 (7.3)		5.6	5.6	
2004–2007	10,492 (11.8)	1999 (25.4)		23.0	23.0	
2008–2011	24,265 (27.3)	2589 (32.9)		32.8	32.8	
2012–2016	35,910 (40.5)	1905 (24.2)		27.0	27.0	
2017–2018	15,830 (17.8)	808 (10.3)		11.7	11.7	

SMD, standardized mean difference; NA, not applicable; OGLDs: oral glucose-lowering drugs; SBP, systolic blood pressure; DBP, diastolic blood pressure; HDL-C, high-density lipoprotein cholesterol; LDL-C: low-density lipoprotein cholesterol; ACR, albumin to creatinine ratio; eGFR, estimated glomerular filtration rate; DPP-4is: dipeptidyl-peptidase 4 inhibitors; TZDs: thiazolidinediones; AGIs: alpha-glucosidase inhibitors; RASi: renin angiotensin system inhibitors.

## Data Availability

Data is contained within the article and Supplementary Material.

## Supplementary Materials

The following supporting information can be downloaded at: https://www.mdpi.com/article/10.3390/ph15091140/s1. Table S1. Definitions of outcomes and covariates. Table S2. Characteristics of metformin and non-metformin users during the observational period in the register-based cohort. Table S3. Characteristics of 100,810 patients in the population-based cohort with propensity-score overlap-weighting (PS-OW). Figure S1. Time frame definitions in the population-based cohort. Figure S2. Study design and patient selection in the register-based cohort. Figure S3. Associations of daily mean dose of metformin with all-cause mortality and MACE by eGFR categories in the population-based cohort.
