# Supplementary material for: Attenuated Risk Association of End-Stage Kidney Disease with Metformin in Type 2 Diabetes with eGFR Categories 1–4
Source: Pharmaceuticals (Basel). 2022 Sep 13;15(9):1140. doi: 10.3390/ph15091140 (PMC9505840; doi:10.3390/ph15091140)
Supplement: Supplementary file 1 [file pharmaceuticals-15-01140-s001.zip › pharmaceuticals-1801784-supplementary.pdf]

Supplemental Material

Figure S1. Time frame definitions in the population-based cohort 2

Figure S2. Study design and patient selection in the register-based cohort 3

Table S1. Definitions of outcomes and covariates 4

Table S2. Characteristics of metformin and non-metformin users during the observational period in the register-based cohort 5

Table S3. Characteristics of 100,810 patients in the population-based cohort with propensity-score overlap-weighting (PS-OW) 6

Figure S3. Associations of daily mean dose of metformin with all-cause mortality and MACE by eGFR categories in the population-based cohort 7

A The new-metformin versus other-OGLDs design

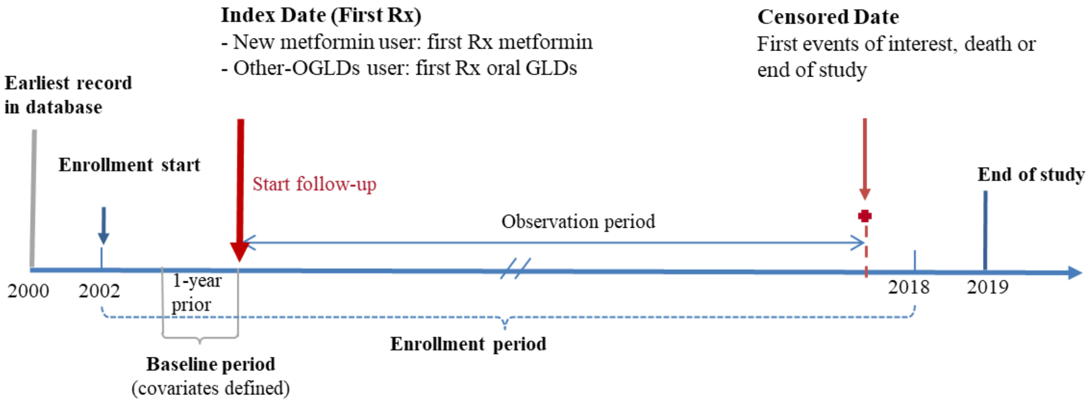

Covariates measured at enrolment
- Sex, age, year of diagnosis, cancer history
- Non-laboratory-based risk factors: Family history, smoking, body mass index, systolic and diastolic blood pressure, and waist circumference

Baseline covariates 1 year prior to index year of start of prescription
- Laboratory-based tests: HbA1c, lipids (triglyceride, total cholesterol, HDL-C and LDL-C), urinary ACR, blood haemoglobin and eGFR
- Comorbidities (CVD and cancer), medications (glucose lowering drugs, RAS inhibitors and statins)

Covariates available at observation period

B The new-metformin versus non-GLDs design

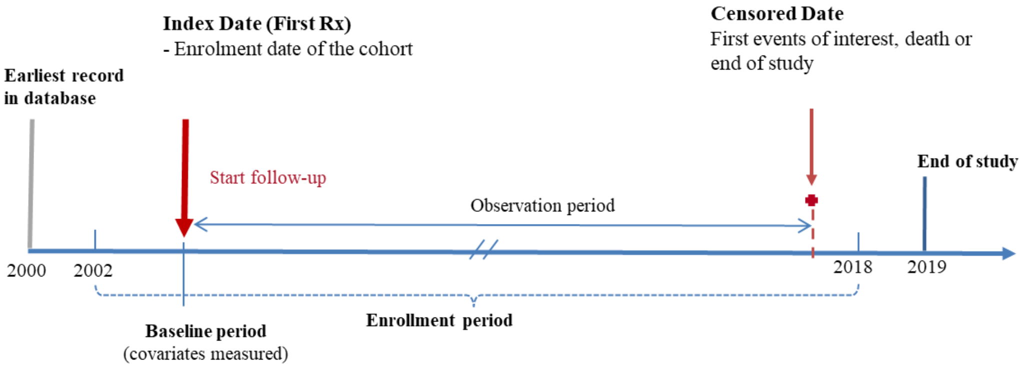

Covariates measured at enrolment
- Age, sex, disease duration, cancer history
- Non-laboratory-based risk factors: family history, smoking, body mass index, systolic and diastolic blood pressure, and waist circumference
- Laboratory-based risk factors: HbA1c, lipids (triglyceride, total cholesterol, HDL-C and LDL-C), urinary albumin-to-creatinine ratio (ACR), blood haemoglobin, estimated glomerular filtration rate (eGFR)
- prevalent use of medications (glucose lowering drugs, RAS inhibitors and statins)

Covariates available during observation period
- Laboratory tests, comorbidities (CVD and cancer), medications

Figure S1. Time frame definitions in the population-based cohort

In the new-metformin versus other-OGLDs design (**A**), the index date was defined as the earliest date of dispensing of metformin or other oral glucose-lowering drugs (OGLDs) after enrolment to the cohort. The follow-up ended at the earliest date of first event of MACE or ESKD or all-cause mortality or censor date (December 31, 2019). In the new-metformin versus non-GLDs design (**B**), the index date was defined as the date of enrolment to the cohort.

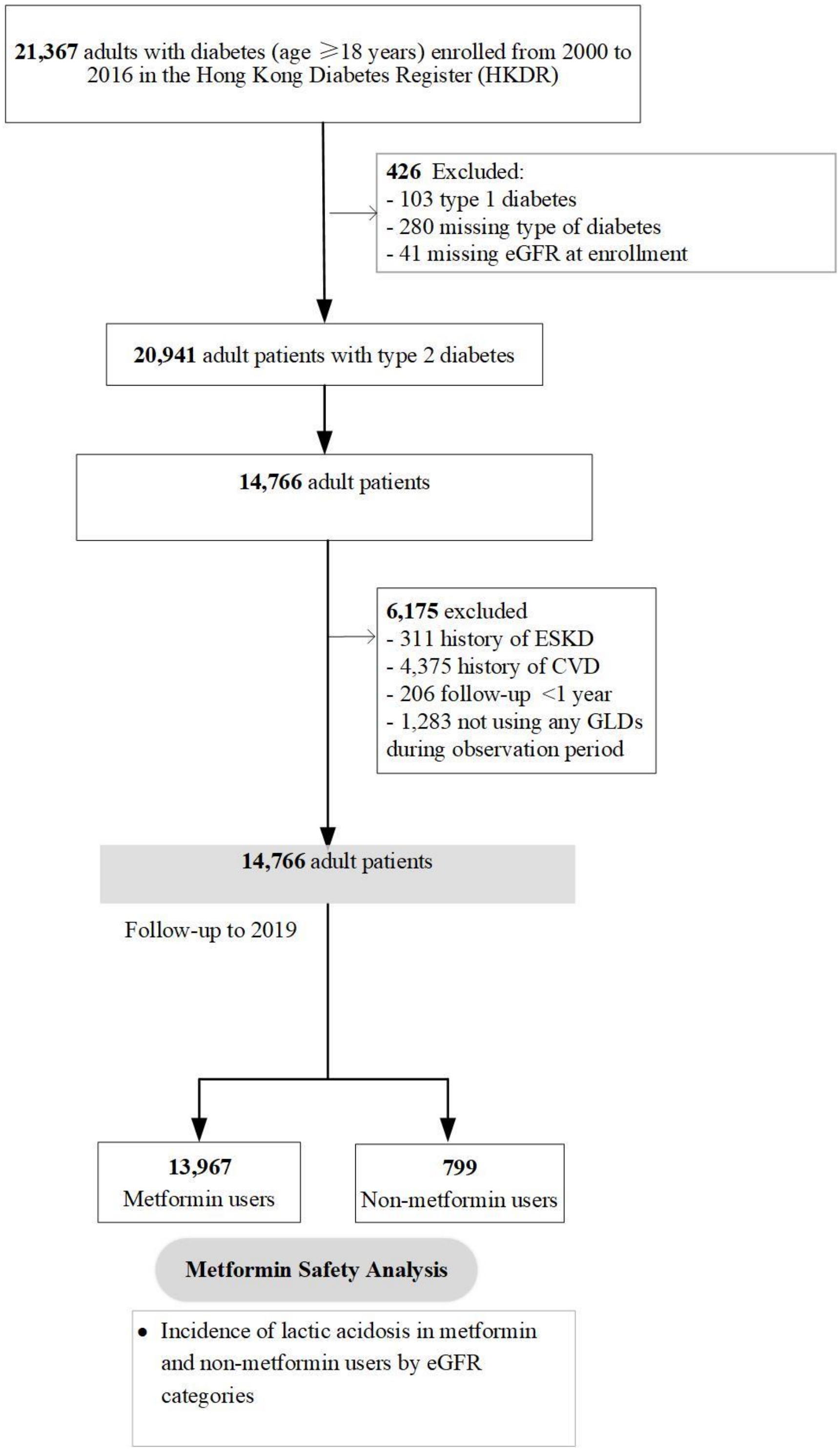

Figure S2. Study design and patient selection in the register-based cohort

Table S1. Definitions of outcomes and covariates

Variables	Definition
All-cause mortality	ICD-10 cause of death from Hong Kong Death Register<br>Cause of death: cardiovascular (CV) death (I00-I99, F01, G45), cancer (C00-C97), kidney disease (N17-N19), pneumonia (J12-J18), others (all other codes)
Major adverse cardiovascular events (MACE)	Nonfatal myocardial infarction (ICD-9 code: 410), non-fatal stroke (ICD-9 code: 430, 431, 434 and 436), and CV-death.
End-stage kidney disease (ESKD)	eGFR < 15 mL/min/1.73 m^2 on at least two occasions at least 90 days apart and that is not associated with acute kidney injury (AKI). eGFR values contained within AKI episodes were first removed (top 15 ICD-9 code 584).<br>Peritoneal dialysis (procedure code: 54.98)<br>Transplant of kidney (procedure code: 55.6)<br>Complications of transplanted kidney (996.81)<br>Persons with a condition influencing their health status; organ or tissue replaced by transplant; kidney (V42.0)
Acute myocardial infarction (AMI)	ICD-9 code: 410
Ischemic heart disease (IHD)	ICD-9 code: 410-414
Heart failure	ICD-9 code: 428
Stroke	ICD-9 code: 430-434, 436
Cardiovascular diseases (CVD)	IHD; stroke; diabetes with peripheral circulatory disorders (250.7); gangrene (785.4); peripheral angiopathy in diseases classified elsewhere (443.81); peripheral vascular disease, unspecified (443.9); 2); other (peripheral) vascular shunt or bypass (procedure code: 39.29); insertion of non-drug-eluting peripheral vessel stent(s) (procedure code: 39.90); amputation of lower limb (procedure code of 84.1)); other procedure codes (surgical revascularization: 38.08, 38.18, 38.38, 38.48, 38.68, 38.88, 39.25, 39.49, 39.56, 39.57, 39.58, 39.59, 39.99; endovascular revascularization: 00.55, 17.56, 39.50, 39.79)
Cancer	ICD-9 code: 140-208
Estimated glomerular filtration rate (eGFR)	The serum creatinine-based CKD-Epidemiological Collaboration (CKD-EPI) Equation
eGFR categories	G1: eGFR >90 mL/min/1.73 m^2; G2: eGFR 60-90 mL/min/1.73 m^2; G3: eGFR 30-59 mL/min/1.73 m^2; G3a: eGFR 45-59 mL/min/1.73 m^2; G3b: eGFR 30-44 mL/min/1.73 m^2; G4: eGFR 15-29 mL/min/1.73 m^2
**Medications**	Based on the Anatomical Therapeutic Chemical (ATC) code
Glucose lowering drugs (GLDs)	
Metformin	ATC: A10BA
**Other-GLDs**	
Insulin	ATC: A10A
Sulfonylureas	ATC:A10BB
Thiazolidinediones	ATC: A10BG
DPP-4is	dipeptidyl peptidase 4 inhibitors, ATC: A10BH
AGIs	alpha-glucosidase inhibitors, ATC: A10BF
GLP-1RAs	glucagon-like peptide-1 (GLP-1) receptor analogues, ATC: A10BJ
SGLT2is	sodium-glucose co-transporter 2 (SGLT2) inhibitors, ATC: A10BK
Other medications	
Statin	HMG CoA reductase inhibitors (ATC: C10AA), simvastatin (ATC: C10AA01), atorvastatin (ATC: C10AA05), rosuvastatin (ATC: C10AA07), fluvastatin (ATC: C10AA04), pravastatin (ATC: C10AA03), lovastatin (ATC: C10AA02)
Renin-angiotensin system inhibitors (RASi)	ACE inhibitors, plain (ATC: C09AA), ACE inhibitors and diuretics (ATC: C09BA), Angiotensin II receptor blockers (ARBs), plain (ATC: C09CA), Angiotensin II receptor blockers (ARBs) and diuretics (ATC: C09DA), Angiotensin II receptor blockers (ARBs) and calcium channel blockers (ATC: C09DB)

ICD, the International Classification of Diseases

Table S2. Characteristics of metformin and non-metformin users during the observational period in the register-based cohort

Characteristics	Metformin users	Non-metformin users
N (%)	13967 (94.6)	799 (5.4)
Men, %	7181 (51.4)	476 (59.6)
Age, years	58.3 (10.7)	64.8 (11.7)
Duration of diabetes, years	6.6 (6.8)	8.1 (7.6)
Family history of diabetes	7705 (55.2)	305 (38.2)
Smoking status		
Non-smoker	9831 (70.4)	505 (63.2)
Ever-smoker	2386 (17.1)	202 (25.3)
Current-smoker	1750 (12.5)	92 (11.5)
Body Mass Index, kg/m^2	25.8 (4.2)	25.1 (4.2)
SBP, mmHg	133.2 (18.1)	139.2 (21.1)
DBP, mmHg	76.7 (10.6)	75.2 (11.6)
Waist, cm	88.3 (10.8)	87.2 (11.2)
Triglyceride, mmol/L	1.7 (1.4)	1.8 (1.8)
Total Cholesterol, mmol/L	4.8 (0.9)	4.9 (1.1)
HDL-C, mmol/L	1.3 (0.4)	1.4 (0.4)
LDL-C, mmol/L	2.7 (0.9)	2.8 (1.0)
Urinary ACR, mg/mmol		
<3	9082 (65.0)	305 (38.2)
3-30	3714 (26.6)	211 (26.4)
>30	1171 (8.4)	283 (35.4)
HbA1c, %	7.5 (1.6)	7.4 (1.8)
eGFR, mL/min/1.73m^2	84.9 (18.7)	56.2 (27.0)
Haemoglobin, gm/dL	13.7 (1.5)	13.0 (1.8)
History of cancer, %	696 (5.0)	75 (9.4)
History of medications, %		
Insulin	2272 (16.3)	219 (27.4)
Sulfonylurea	8120 (58.1)	557 (69.7)
DPP-4is	665 (4.8)	52 (6.5)
TZDs	534 (3.8)	30 (3.8)
AGIs	387 (2.8)	33 (4.1)
Not on any GLDs	2491 (17.8)	181 (22.7)
Statin	4082 (29.2)	245 (30.7)
RASi	5465 (39.1)	393 (49.2)
Period of index year, %		
<2003	1913 (13.7)	179 (22.4)
2004-2007	1993 (14.3)	123 (15.4)
2008-2011	5848 (41.9)	287 (35.9)
2012-2016	4213 (30.2)	210 (26.3)

SBP, systolic blood pressure; DBP, diastolic blood pressure; HDL-C, high-density lipoprotein cholesterol; LDL-C: low-density lipoprotein cholesterol; ACR, albumin to creatinine ratio; eGFR, estimated glomerular filtration rate; DPP-4is: dipeptidyl-peptidase 4 inhibitors; TZDs: thiazolidinediones; AGIs: alpha-glucosidase inhibitors; RASi: renin angiotensin system inhibitors.

Table S3. Characteristics of 100,810 patients in the population-based cohort with propensity-score overlap-weighting (PS-OW)

Characteristics	New-metformin versus non-GLDs users					
	Before PS-OW			After PS-OW		
	New-metformin	Non-GLDs	SMD	New-metformin	Non-GLDs	SMD
n (%)	70539	30271		70539	30271	
Men, %	31757 (45.0)	12976 (42.9)	0.043	42.7	42.7	<0.001
Age, years	59.8 (10.9)	64.9 (11.4)	0.455	63.0 (11.5)	63.0 (10.3)	<0.001
Duration of diabetes, years	1.9 (4.1)	1.3 (3.1)	0.177	1.4 (3.5)	1.4 (3.1)	<0.001
Family history of diabetes	26348 (37.4)	9453 (31.2)	0.129	33.4	33.4	<0.001
Smoking status			0.113			<0.001
Non-smoker	55736 (79.0)	24142 (79.8)		80.2	80.2	
Ever-smoker	7931 (11.2)	4022 (13.3)		12.3	12.3	
Current-smoker	6872 (9.7)	2107 (7.0)		7.5	7.5	
Body Mass Index, kg/m^2	26.5 (4.2)	25.9 (4.1)	0.130	26.2 (4.2)	26.2 (4.0)	<0.001
SBP, mmHg	133.2 (13.1)	133.6 (14.2)	0.034	133.3 (14.1)	133.3 (13.1)	<0.001
DBP, mmHg	75.3 (8.5)	74.2 (9.4)	0.117	74.7 (9.3)	74.7 (8.7)	<0.001
Waist, cm	90.4 (10.2)	89.5 (10.1)	0.098	90.0 (10.2)	90.0 (9.9)	<0.001
Triglyceride, mmol/L	1.8 (1.5)	1.6 (1.0)	0.186	1.6 (1.2)	1.6 (1.1)	<0.001
Total Cholesterol, mmol/L	5.2 (1.0)	5.0 (1.0)	0.202	5.0 (1.0)	5.0 (1.0)	<0.001
HDL-C, mmol/L	1.3 (0.3)	1.3 (0.4)	0.176	1.3 (0.4)	1.3 (0.4)	<0.001
LDL-C, mmol/L	3.1 (0.9)	2.9 (0.8)	0.197	3.0 (0.9)	3.0 (0.9)	<0.001
Urinary ACR, mg/mmol			0.044			<0.001
<3	52049 (73.8)	22918 (75.7)		75.9	75.9	
3-30	15936 (22.6)	6340 (20.9)		20.9	20.9	
>30	2554 (3.6)	1013 (3.3)		3.1	3.1	
HbA1c, %	7.4 (1.7)	6.4 (0.7)	0.834	6.5 (0.8)	6.5 (0.6)	<0.001
eGFR, mL/min/1.73m^2	86.2 (16.8)	81.4 (17.8)	0.280	83.3 (17.7)	83.3 (16.1)	<0.001
Haemoglobin, gm/dL	13.9 (1.5)	13.7 (1.5)	0.136	13.8 (1.5)	13.8 (1.5)	<0.001
History of cancer, %	2312 (3.3)	1637 (5.4)	0.105	4.7	4.7	<0.001
History of medications, %						
Statin	12143 (17.2)	9837 (32.5)	0.359	27.9	27.9	<0.001
RASi	12905 (18.3)	7987 (26.4)	0.195	24.3	24.3	<0.001
Period of index year, %			0.625			<0.001
<2003	5123 (7.3)	291 (1.0)		1.6	1.6	
2004-2007	7949 (11.3)	1151 (3.8)		5.7	5.7	
2008-2011	20479 (29.0)	5831 (19.3)		23.8	23.8	
2012-2016	27254 (38.6)	13480 (44.5)		45.7	45.7	
2017-2018	9734 (13.8)	9518 (31.4)		23.2	23.2	

SMD, standardized mean difference; NA, not applicable; GLDs: glucose-lowering drugs; SBP, systolic blood pressure; DBP, diastolic blood pressure; HDL-C, high-density lipoprotein cholesterol; LDL-C: low-density lipoprotein cholesterol; ACR, albumin to creatinine ratio; eGFR, estimated glomerular filtration rate; RASi: renin angiotensin system inhibitors.

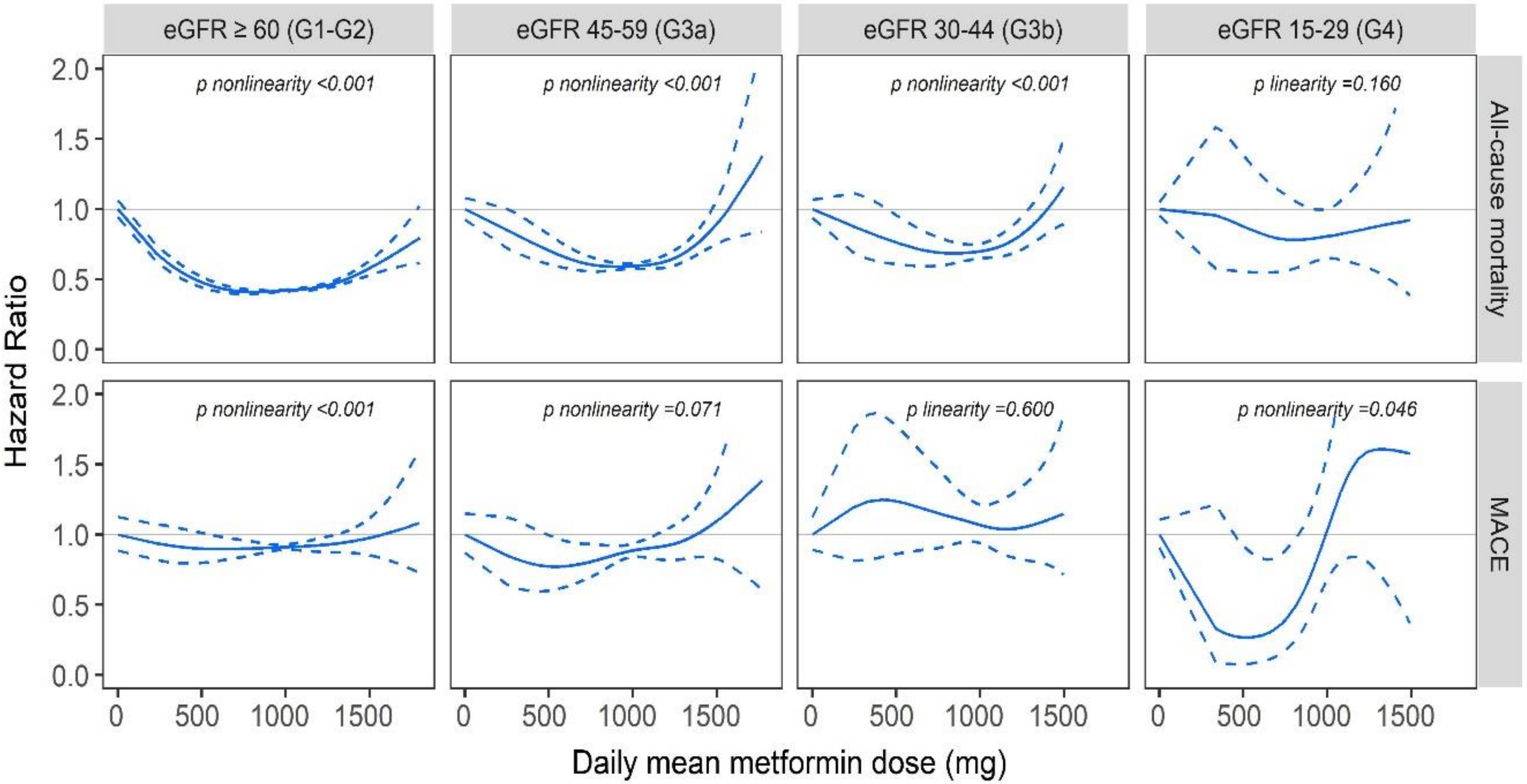

Figure S3. Associations of daily mean dose of metformin with all-cause mortality and MACE by eGFR categories in the population-based cohort

Penalized spline curve analyses with 3-knots were performed (n=96,643) using Cox-model with time-varying mean daily dose of metformin exposure, adjusted for confounding effects due to age, sex, disease duration, cardiometabolic risk factors and cancer history at enrolment as well as time-varying covariates including HbA1c, lipids, comorbidities (CVD and cancer), and use of diabetes-related medications (insulin, oral glucose-lowering drugs, RAS inhibitors and statins) during observation.
